# *H. pylori* isolates with amino acid sequence polymorphisms as presence of both HtrA-L171 & CagL-Y58/E59 increase the risk of gastric cancer

**DOI:** 10.1186/s12929-019-0498-9

**Published:** 2019-01-05

**Authors:** Yi-Chun Yeh, Hsin-Yu Kuo, Wei-Lun Chang, Hsiao-Bai Yang, Cheng-Chan Lu, Hsiu-Chi Cheng, Ming-Shiang Wu, Bor-Shyang Sheu

**Affiliations:** 1 Department of Internal Medicine, National Cheng Kung University Hospital, College of Medicine, National Cheng Kung University, Tainan, Taiwan; 2 Institute of Clinical Medicine, College of Medicine, National Cheng Kung University, Tainan, Taiwan; 3 Department of Pathology, National Cheng Kung University Hospital, College of Medicine, National Cheng Kung University, Tainan, Taiwan; 4 Department of Pathology, Ton Yen General Hospital, Hsin-Chu, Taiwan; 5 Department of Internal Medicine, Tainan Hospital, Ministry of Health and Welfare, 125 Chuang Shan Road, Tainan, Taiwan; 6 Department of Internal Medicine, National Taiwan University Hospital, College of Medicine, National Taiwan University, Taipei, Taiwan

**Keywords:** Gastric cancer, *H. pylori*, HtrA, CagL, Type IV secretory system

## Abstract

**Background:**

*H. pylori* CagL-Y58/E59 increase gastric cancer risk by stronger binding with integrin to faciliate type IV secretory system (T4SS). *H. pylori* can secrete high temperature requirement A (HtrA) to mediate E-Cadherin cleavage for gastric epithelial junction disruption, so *H. pylori* CagL can adhere to integrin located on basolateral side of epithelium. The study test whether *H. pylori* HtrA amino acid polymorphisms can increase gastric cancer risk synergistically with CagL-Y58/E59.

**Methods:**

One-hundred and sixty-four *H. pylori*-positive patients, including 71 with non-ulcer dyspepsia (NUD), 63 with peptic ulcers (PU), and 30 with gastric cancers (GC), were enrolled to receive upper gastrointestinal endoscopy to obtain gastric biopsies for *H. pylori* culture and histology by the updated Sydney system. Each isolate was screened for *htrA* & cagL genotype by polymerase chain reaction and HtrA & CagL-Y58/E59 amino acid sequence polymorphisms by sequencing.

**Results:**

The prevalence rates of *htrA & cagL* gene were both 100%. The HtrA amino acid sequence polymorphisms were not different between NUD and PU. The *H. pylori* isolates of GC had higher rates of HtrA residue 171 as leucine than those of NUD (73.3% vs. 50.7%, *P* = 0.036, OR[95%CI] = 2.7[1.1–6.8]). The risk of the *H. pylori*-infected subjects to get gastric cancer was increased up to 15.4-fold, if the infected isolates had presence of both HtrA-L171 and CagL-Y58/E59 (*P* < 0.001).

**Conclusions:**

The *H. pylori* isolates *of* gastric cancer subjects had a higher rate of HtrA-L171. *H. pylori* isolates with presence of both HtrA-171 & CagL-Y58/E59 can synergistically increase the risk of gastric cancer.

**Electronic supplementary material:**

The online version of this article (10.1186/s12929-019-0498-9) contains supplementary material, which is available to authorized users.

## Introduction

*Helicobacter pylori* infection leads to chronic gastritis, peptic ulcers and gastric adenocarcinoma [1, 2]. *H. pylori* isolates can display extensive diversities in polymorphisms of virulence factor genes, which may determine an increased risk to have gastrointestinal disorders [3–5]. To search the virulence factors of the infected *H. pylori* isolates can thus identify the risky groups for earlier treatment to control the adverse outcome, particularly gastric cancer.

Triple positive *cagA-vacA-babA2 H. pylori* infection increases the risks of peptic ulcer and gastric cancer in the Western countries [6]. However, in Taiwan, the incidence of triple positive infection is near 100%, so such genomic polymorphisms are not related to the different clinical outcomes [7–10]. CagL, as a component of type VI secretion system (T4SS), binds to integrins α5β1 at the basolateral side of host gastric epitheliums to facilitate CagA translocation for carcinogenesis [11]. The *H. pylori* isolates with CagL amino acid polymorphisms as Y58/E59 exploit higher integrin α5β1 to carry 4.6-fold risk increase of gastric cancer [4]. Studies analyzed Asian and non-Asian subject cohorts were consistent with our finding to show that CagL-E59 was associated with gastric cancer [12–14]. Moreover, we previous reported *H. pylori* CagL-Y58/E59 can prime higher integrin α5β1 in adverse pH condition to enhance hypochlorhydria vicious cycle for gastric carcinogenesis [15]. However, some results contrasted with those finding in 26,695 and P12 strains [16, 17]. In addition, It is still nearly 50% of gastric cancer *H. pylori* isolates without CagL-Y58/E59, and additional virulence factors to increase risk of gastric cancers are in need of further validation.

High-temperature requirement A (HtrA) protein is a chaperone and serine protease. *H. pylori* HtrA consists of signal domain, serine protease domain, PDZ-1 and PDZ-2 domain [18, 19]. The secreted HtrA of *H. pylori* opened tight junctions and adherence junctions via cleaving occludin, claudin-8, and the extracellular domain of E-cadherin, and consequently *H. pylori* can across epithelial monolayer to the basolateral membranes to interact with integrin α5β1 for T4SS injection of CagA [18, 20]. The residue S221 has been shown as the active site [18, 21]. Moreover, a second hot-spot site is around Q81 according to iPred interface prediction and is confirmed by catalytic activity of Q81A mutation. In addition, some charged residues at the HtrA surface, such as D165, D168 and D260, also display important roles for HtrA activity [22]. Additionally, S164, S166, N208 and K328 are the presumed binding sites of HtrA inhibitor, and their mutations loss proteolytic activity against E-cadherin [19].

The *H. pylori* isolates from worldwide are highly variable in nucleotide sequence of *htrA* gene, and had strain-specific difference in the HtrA cleavage E-cadherin [23]. This study thus validated whether *htrA* genopositivity or any specific polymorphisms of HtrA amino acid sequence can determine the risk with clinical outcomes after *H. pylori* infection, especially in non CagL-Y58/E59 status. In addition, we validated whether any specific HtrA amino acid sequences synergistically increase the risk of gastric cancer with CagL-Y58/E59. Our data shall be original to suggest HtrA amino acid polymorphisms of *H. pylori* isolates as virulence factor of gastric cancer. The screening strategy for specific HtrA sequences of *H. pylori* isolates will be promising to select risky group for early *H. pylori* eradication to improve gastric cancer control.

## Materials & methods

### Patients and collection of H. pylori isolates

*H. pylori* strains were obtained from the gastric biopsy of *H. pylori*-infected patients who underwent upper gastrointestinal endoscopy at National Cheng Kung University Medical Center, Tainan, Taiwan. We proposed the rate of HtrA amino acid polymorphisms in non-cancer strains was 50%. The number of *H. pylori* strains from patients with gastric cancers, non-ulcer dyspepsia, and peptic ulcers was at a 1:2:2 ratio. The total number of strains required was 123 to detect a 30% difference in the polymorphism rates between cancer strains and non-cancer strains with a two-sided α value of 0.05 and a power of 80% (β = 0.20). Assuming a screening failure rate of 20%, 148 strains at least were needed. A total of 164 isolates were obtained from 71 non-ulcer dysplasia (NUD), 63 peptic ulcer (PU), and 30 gastric adenocarcinoma (GC) patients.

In each patient, the endoscopic diagnosis and topographic gastric biopsy for *H. pylori* related pathology were reviewed. Biopsies were stained with haematoxylin and eosin, as well as with modified Giemsa stains, to evaluate the *H. pylori*-related histological features and to grade severity according to the updated Sydney system [24]. The acute inflammatory score (AIS, range 0–3), chronic inflammation score (CIS, range 0–3), the *H. pylori* density (HPD) for each specimen was scored as our previous studies: in range of 0–5 for each biopsy specimen.

None of the cases have used with antibiotics or proton pump inhibitor before endoscopy. Each patient has provided blood sampling before endoscopy to obtain serum for pepsinogen and gastrin assay.

### *H. pylori isolate* DNA extraction, PCR and sequencing for htrA & cagL gene

Genomic DNA of *H. pylori* was extracted by using the Genomic DNA Purification Kit (ThermoFisher Scientific). The extracted DNA of each isolate was subjected to PCR to amplify the *htrA* genes using paired primers: htrA_F (5′- GCA TCG GGA TGA TTT TAA CG-3′) and htrA_R (5′-AAA CAA CGC TCG TTT GTT TG-3′) (Genomics, Taiwan). The PCR mixtures were made in a volume of 50 μl containing 200 ng of DNA, 0.2 mM of primers and 25 μl of GoTaq ® Green Master Mix (Promega, Madison, WI). The PCR reaction was performed with a thermal cycler (2720 thermal cycler, Applied Biosystems, Foster City, CA) under 94 °C for 5 min, 30 cycles of 94 °C for 30 s, 60 °C for 30 s, and 72 °C for 1 min, and then followed by a final elongation step at 72 °C for 10 min. The mixture was stored at 4 °C. The PCR products were separated by 2% agarose gel electrophoresis and examined under UV illumination [4, 25]. The genotype of cagL was applied as the method as used in our previous literature [4].

### htrA & cagL-gnosequencing for translating into amino acid sequences

The amplified products of the *htrA* were then subjected to the direct sequencing (Genomics, Taiwan). The nucleotide sequence identities were compared with sequences deposited in the GenBank database by BLAST program at the National Center for Biotechnology Information. Amino acid sequences of HtrA were predicted by the standard code, and aligned using GeneDoc (version 2.7.000). The amplified product to assess the CagL-Y58/E59 was applied as in our previous publication [4]. The 164 *htrA* and 97 *cagL* gene sequences were deposited in the GenBank, and the accession numbers were showed in Additional files 1 and 2, respectively.

### Serum pepsinogen and gastrin by enzyme-linked immuno-sorbent assay (ELISA)

Blood samples were collected and centrifuged at 3000 rpm for 15 min at 4 °C. Sera were obtained and frozen immediately at − 80 °C until analysis. These collected sera were then measured for the levels of pepsinogen I and II (Biohit Oyj, Helsinki, Finland), and gastrin using ELISA kit (BlueGene Biotech, Shanghai, China) according to the manufacturer’s specifications [15, 26].

### Statistics

The statistical analysis was performed with the SPSS software (SPSS 12, Chicago, IL, USA). The χ^2^ test was used to validate the correlation among gender, *htrA* prevalence rates, and amino acid sequence polymorphisms. The *t* test was used to validate age and serum levels of pepsinogen I, pepsinogen II and gastrin. The Mann-Whitney U test was applied to analyze the differences in histological severity. *P* value < 0.05 was considered significant with two-tailed analysis.

## Results

### The association of HtrA amino acid polymorphism with the clinical diagnosis

Of the 164 *H. pylori*-positive dyspeptic patients, endoscopic diagnoses included 71 with NUD, 63 with PU, and 30 with GC. The enrolled subjects with GC had higher mean age than those with NUD or PU (59.2 ± 13.2 vs. 47. 3 ± 12.3 vs. 49.5 ± 13.2, respectively, *P* < 0.001). Males had higher rates of PU than females as compared to NUD subjects (61.9% vs. 31%, *P* < 0.001).

The prevalence of *htrA* gene of 164 collected isolates was 100% (). The HtrA amino acid sequence of our isolates had more than 95% homology. Based on these predicted amino acid sequences, there were 7 residues of HtrA with more than 10% variation in these isolates, including residue 6F/L, 25 N/S, 68S/N, 171 L/S, 303 V/I, 312A/V, and 382 T/A or V (Table 1).

**Table 1:** The polymorphisms of HtrA amino acid sequence among isolate from patients with different clinical diagnosis

Residue n (%)	NUD (*n* = 71)	PU (*n* = 63)	GC (*n* = 30)	*P* value ^a^ OR (95% CI)	*P* value ^b^ OR (95% CI)
6 F	40 (56.3)	39 (61.9)	19 (63.3)	0.513 1.3 (0.6–2.5)	0.515 1.3 (0.6–3.2)
25 N	20 (28.2)	16 (25.4)	11 (36.7)	0.718 0.9 (0.4–1.9)	0.397 1.5 (0.6–3.6)
68 S	11 (15.5)	9 (14.3)	8 (26.7)	0.845 0.9 (0.4–2.4)	0.189 2.0 (0.7–5.6)
171 L	36 (50.7)	38 (60.3)	22 (73.3)	0.264 1.5 (0.7–2.9)	0.036 2.7 (1.1–6.8)
303 V	52 (73.2)	48 (76.2)	24 (80.0)	0.695 1.2 (0.5–2.6)	0.472 1.5 (0.5–4.1)
312 A	44 (62.0)	39 (61.9)	19 (63.3)	0.994 1.0 (0.5–2.0)	0.897 1.1 (0.4–2.6)
382 T	16 (22.5)	11 (17.5)	9 (30.0)	0.465 0.7 (0.3–1.7)	0.427 1.5 (0.6–3.8)

Abbreviations: *NUD* non-ulcer dyspepsia, *PU* peptic ulcer, *GC* gastric cancer, *OR* odds ratio, *95% CI* 95% confidence interval. The *P* value was determined by χ^2^ test. ^a^ indicated significance with *P* < 0.05 of such parameter between NUD and PU; ^b^ between NUD and GC

**Fig. 1:**
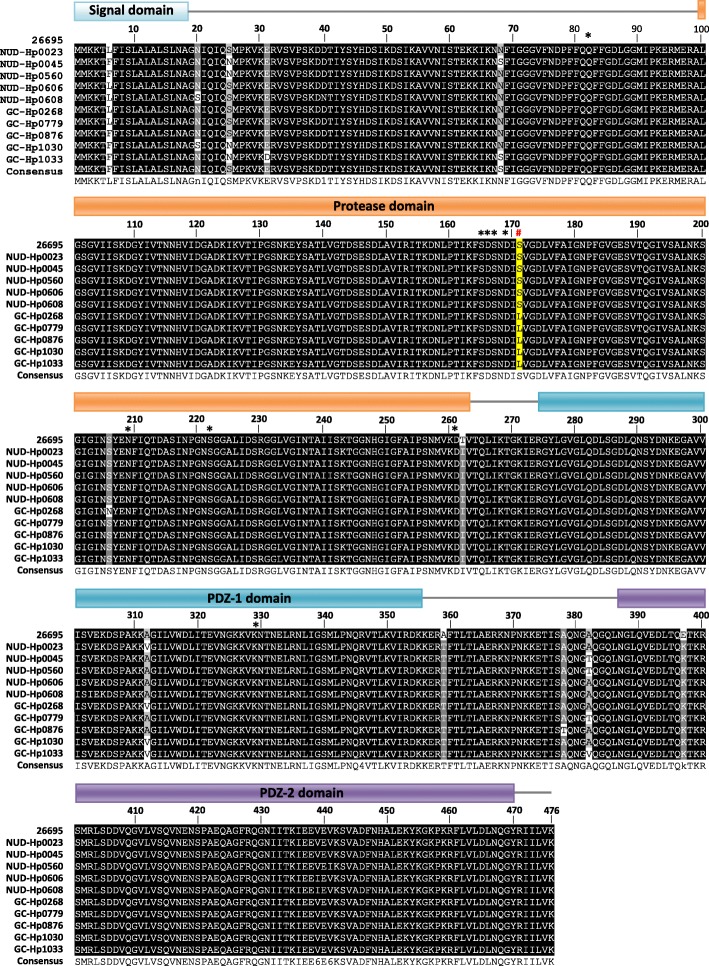
The amino acid sequences of HtrA among 26,695 and 10 clinical isolates are listed, including 5 NUD isolates (NUD-Hp0023, NUD-Hp0045, NUD-Hp0560, NUD-Hp0606, NUD-Hp0608), and 5 GC isolates (GC-Hp0268, GC-Hp0779, GC-Hp0876, GC-Hp1030, GC-Hp-1033). The “*” indicates the active residues 81, 164, 165, 166, 168, 208, 221, 260 and 328 (according to HtrA sequence from NCBI, residue 81, 164, 165, 166, 168, 208, 221, 260 and 328 should be residue 82, 165, 166, 167, 169, 209, 222, 261 and 329, respectively), and the “#” indicates sequences with variations at the residue 171

Among these 7 variant residues, 6F/L was located at signal domain, 171 L/S was located at protease domain, as well as 303 V/I and 312A/V were located at PDZ-1 domain (Fig. 1). We analyzed the correlation between HtrA amino acid polymorphisms and clinical diagnosis showed in Table 1. The active sits S211 and functional regulation residues at Q81, S164, D165, S166, D168, N208, D260 and K328 were all conserved among all isolates (Fig. 1).

There were no difference of HtrA amino acid sequence polymorphisms between NUD and PU subjects (*P* > 0.05). However, isolates from GC had significantly higher rates of amino acid sequence 171 as leucine than the isolates from the NUD subjects (*P* = 0.036, OR[95% CI] = 2.7[1.1–6.8]) (Table 1). There were no difference in the present rate of F6, N25, S68, V303, A312, and T382 between NUD and GC subjects (*P* > 0.05).

### Combined HtrA-L171 and CagL-Y58/E59 between gastric cancer and NUD subjects

Our previous study showed about 50% gastric cancer isolates with CagL-Y58/E59 sequence, and subjects infected with *H. pylori* with this sequence had increased risk of gastric cancer [4]. However, there were nearly 50% gastric cancer isolates without bearing CagL-Y58/E59. We thus further examined whether *H. pylori* infection carrying with HtrA-L171 correlates the risk of gastric cancer if subjects infected with a CagL-Y58/E59 absent *H. pylori*.

In Table 2 showed as CagL-Y58/E59 absence, the prevalence of HtrA-L171 was 86.7% in GC isolates, and significantly higher than in NUD isolates (50.9%) (*P* = 0.013; OR[95%CI] = 6.3[1.3–30.4]). We further analyzed whether combined HtrA-L171 and CagL-Y58/E59 have synergistic effect on the risk of gastric cancer development. In Table 2, the odds ratio was higher for those infected by *H. pylori* bearing combined HtrA-L171 and CagL-Y58/E59, and the risk could be even higher than as *H. pylori* with HtrA-L171 alone or CagL-Y58/E59 alone.

**Table 2:** The comparison of the GC risk as *H. pylori* bearing with different HtrA-L171 and CagL-Y58/E59 status

HtrA-L171	CagL-Y58/E59	NUD n (%)	GC n (%)	*P* value	OR (95% CI)
-	-	27 (49.1)	2 (13.3)	0.013	6.3 (1.3–30.4)
-	+	28 (50.9)	13 (86.7)		
-	-	27 (81.8)	2 (25.0)	0.002	13.5 (2.2–84.0)
+	-	6 (18.2)	6 (75.0)		
-	-	27 (79.4)	2 (20.0)	< 0.001	15.4 (2.7–89.5)
+	+	7 (20.6)	8 (80.0)		

Abbreviations: *NUD* non-ulcer dyspepsia, *GC* gastric cancer, *OR* odds ratio, *95% CI* 95% confidence interval. The *P* value was determined by χ^2^ test

### Gastric histology in NUD with different H. pylori HtrA-L171 & CagL-Y58/E59 status

Table 3 presented the differences in histological features in the antrum and in the corpus among *H. pylori* infection carrying with different *H. pylori* HtrA-L171 & CagL-Y58/E59 status in NUD subjects. Only in the antrum, the CIS was higher in the subjects with *H. pylori* bearing HtrA-L171 alone than in other those infected by *H. pylori* with other HtrA-L171 & CagL-Y58/E59 status (*P* < 0.05).

**Table 3:** The comparison of histopathology among NUD subjects with different status of HtrA-L171 & CagL-Y58/E59 of *H. pylori* infection

Histology score, mean (SD)	HtrA-L171 & CagL-Y58/E59 status				*P* value
	Both absence (*n* = 27)	HtrA-171 L alone (*n* = 28)	CagL-Y58/E59 alone (*n* = 6)	Both presence (*n* = 7)	
Antrum					
AIS	1.44 (0.97)	1.64 (0.78)	1.33 (1.03)	1.29 (0.95)	NS
CIS	2.74 (0.53)	2.96 (0.19)	2.67 (0.82)	2.71 (0.49)	0.039^a^; 0.037^b^
HPD	3.26 (1.26)	3.36 (1.19)	3.17 (1.72)	3.86 (1.07)	NS
Corpus					
AIS	1.00 (0.96)	0.93 (0.98)	1.17 (0.98)	0.57 (0.98)	NS
CIS	2.37 (0.79)	2.32 (0.82)	2.83 (0.41)	2.14 (0.07)	NS
HPD	3.41 (1.37)	3.46 (1.17)	3.67 (1.03)	2.43 (1.62)	NS

Abbreviations: *AIS* acute inflammation score (range 0–3), *CIS* chronic inflammation score (range 1–3); HPD, *H. pylori* density (range 0–5). Statistical analysis was performed by Mann-Whitney U test. ^a^ indicated significance with *P* < 0.05 of such parameter between both absence and HtrA-L171 alone *H. pylori* infection; ^b^ between HtrA-L171 alone and both presence. NS: no significant difference

### Serum gastrin, pepsinogen I & II of NUD with different HtrA-L171 & CagL-Y58/E59

Next we compared the difference of serum markers, including PG I, PG II and gastrin, among NUD subjects with *H. pylori* infection bearing different HtrA-L171 and CagL-Y58/E59 status. In Table 4, the presence of HtrA-L171 alone in the *H. pylori*-infected NUD subjects had higher serum gastrin than those infected with double absence of HtrA-L171 & CagL-Y58/E59 *H. pylori* isolates (96.6 ± 36.8 pg/ml vs. 34.0 ± 26.9 pg/ml, *P* = 0.003).

**Table 4:** The comparison of serum levels of gastrin, PG I and II among NUD subjects with the different status of HtrA-L171 & CagL-Y58/E59 of *H. pylori* infection

Parameters, mean (SD)	HtrA-L171 & CagL-Y58/E59 status				*P* value
	Both absence (*n* = 11)	HtrA-171 L alone (*n* = 7)	CagL-Y58/E59 alone (*n* = 6)	Both presence (*n* = 5)	
Gastrin (pg/ml)	34.0 (26.9)	96.6 (36.8)	60.8 (48.0)	54.6 (20.7)	0.003^a^
PG I (ng/ml)	105.3 (59.2)	112.3 (27.7)	116.8 (29.0)	99.6 (26.1)	NS
PG II (ng/ml)	12.2 (6.1)	11.5 (5.0)	17.4 (8.6)	11.7 (3.9)	NS
PG I/II	9.0 (3.2)	10.6 (3.2)	7.4 (1.8)	9.4 (4.2)	NS

Abbreviations: *PG* pepsinogen. Statistical analysis was performed by t test. ^a^ indicated significance with *P* < 0.05 of such parameter between both absence of HtrA-L171 & CagL-Y58/E59 and HtrA-L171 only *H. pylori* infection. NS: no significant difference

## Discussion

This study surveyed whether the serine protease HtrA of *H. pylori* correlated with the gastrointestinal disorder, particularly gastric cancers after *H. pylori* infection. The *htrA-*genopositive prevalence of *H. pylori* isolates were 100% in Taiwan. Moreover, HtrA active sites were conserved among all *H. pylori* isolates. Our study exhibited that infection of *H. pylori* harboring HtrA-171 as leucine correlated with higher risk of GC. Subjects with such *H. pylori* infection had elevated serum levels of gastrin and more server chronic inflammation in the antrum. Moreover, the risk of the *H. pylori*-infected subjects to get gastric cancer was increased up to 15.4-fold, if the infected isolates had presence of both HtrA-L171 and CagL-Y58/E59.

In the present study, the prevalence of *htrA* gene of *H. pylori* isolations from Taiwan was 100% and consistent with recent report by Tegtmeyer et al. [23]. In addition, our study has applied sequencing to survey the active site 221 and relevant functional regulation sites of HtrA, including residue 81, 164, 165, 166, 168, 208, 260 and 328. Our data showed these residues were conserved in all our isolations and thus confirmed HtrA should be crucial for *H. pylori* survival.

Since the universal presence of HtrA active residues, we further checked whether there was existence of HtrA amino acid sequence polymorphisms to determine the risk of diverse clinical disease outcomes. In Table 1, we demonstrated that isolates bearing HtrA residue L171 had a 2.7-fold risk of gastric cancer development. In our previous report disclosed that *H. pylori* carrying with CagL-Y58/E59 correlated to a 4.6-fold risk of GC. However, still nearly 50% of GC isolates had absence of CagL-Y58/E59 [4]. We thus further validate whether HtrA amino acid sequence can be helpful to identify the gastric cancer risk, especially when the *H. pylori* isolates lack CagL-Y58/E59. In this study, we disclosed *H. pylori* carrying with HtrA-L171 indeed exhibited an increased GC risk as CagL-Y58/E59 absence (Table 2). It reveals that HtrA-L171 has potential as a marker of GC development.

Residue 171 is located at the protease domain of HtrA. Base on the report by Perna and colleagues showing that HtrA mutant S164A, S166A, N208A, and K328A lost their ability to cleave E-cadherin. This observation supports that these residues played relevant roles for the functional regulation of HtrA [19]. L171 is also closed to S164 and S166 (according to NCBI database, S164 and S166 should be S165 and S167 respectively, Fig. 1). Accordingly, it deserves future study to test whether L171 can be a relevant site for the HtrA regulation or whether amino acid change at 171 alters the function of S164 or S166.

Because HtrA mediates disruption of adherence junction via E-cadherin cleavage, *H. pylori* consequently enables to proceed the interaction of CagL and integrin α5β1 at basolateral membrane and then to inject effector protein CagA into host epithelium cells [18, 21]. The study thus checked whether combined HtrA-L171 and CagL-Y58/E59 have synergistic risk on GC. In Table 2, the risk of GC as *H. pylori* carrying with combined HtrA-L171 and CagL-Y58/E59 was higher than as those isolates carrying with HtrA-L171 only or with CagL-Y58/E59 only. The combined effect from stronger E-cadherin cleavage by HtrA-171 and stronger integrin expression for T4SS by CagL-Y58/E59 may explain the risk increment in part.

We previously elaborated the CagL-Y58/E59 infection with corpus shift of integrin α5β1 can cause more severe gastric corpus-predominant injury [4]. Corpus-predominant chronic inflammation reduced acid secretion by parietal cell loss, and facilitates carcinogenetic progression [27]. *H. pylori* with CagL-Y58/E59 can prime more integrin α5β1 to translocate CagA under hypochlorhydria for gastric carcinogenesis [15]. Unlike CagL-Y58/E59 *H. pylori* infection, subjects infected with *H. pylori* harboring HtrA-L171 displayed more severe gastric antrum-predominant inflammation instead (Table 3). It implies that HtrA-L171 mediating carcinogenesis may differ from the process via the loss of mucosal glands in the corpus.

Subjects with HtrA-L171 *H. pylori* infection had elevated serum levels of gastrin (Table 4). Gastrin is an important regulator in gastric acid secretion. Moreover, gastrin can regulate the growth and differentiation of gastric epithelial cells, which might be relevant in the pathogenesis of gastric cancer [28]. Increased serum gastrin levels may result from proton pump inhibitors use, atrophic gastritis, and *H. pylori* infection [29, 30]. Wiedemann et al. reported that the interaction of CagL and integrin αvβ5 activate gastrin promoter through EGFR/Raf/MAP/Erk signaling cascade [31]. In Table 4, however, there was no significant difference in serum levels of gastrin as *H. pylori* carrying with CagL-Y58/E59 compared to other groups.

Inge et al., showed that soluble E-cadherin can activate EGFR and downstream ERK1/2 signaling pathway [32]. Because HtrA mediates cleavage of E-cadherin and generates soluble E-cadherin, whether the soluble E-cadherin generated by HtrA may activate EGFR and consequently activate gastrin promoter or mRNA secretion to contribute GC development deserves further examination.

The limitation to this study is just analysis of isolates from Taiwan. It is in need to further validate in a large number of isolates from Taiwan and other parts, particularly in the western worlds. Furthermore, there is a need to validate whether HtrA-171 polymorphism really exist different ability in protease activity.

## Conclusion

This study showed the Taiwanese *H. pylori* isolates bearing HtrA-L171 associated with increased risk of GC development, which may work individually or collaborate with CagL-Y58/E59. HtrA-L171 may serve to screen out the risk population for *H. pylori* eradication so as to impede the carcinogenesis process.

## Additional files

Additional file 1: Accession numbers of *htrA* gene analyzed in this study. (DOCX 37 kb)

Additional file 2: Accession numbers of *cagL* gene analyzed in this study. (DOCX 28 kb)

## Abbreviations

AIS: Acute inflammatory score
CIS: Chronic inflammation score
ELISA: Enzyme-linked immuno-sorbent assay
GC: Gastric cancers
*H. pylori*: *Helicobacter pylori*
HPD: *H. pylori* density
HtrA: High temperature requirement A
NUD: Non-ulcer dyspepsia
PCR: Polymerase chain reaction
PU: Peptic ulcers
T4SS: Type IV secretory system
