# Supplementary material for: H. pylori isolates with amino acid sequence polymorphisms as presence of both HtrA-L171 & CagL-Y58/E59 increase the risk of gastric cancer
Source: J Biomed Sci. 2019 Jan 5;26:4. doi: 10.1186/s12929-019-0498-9 (PMC6321681; doi:10.1186/s12929-019-0498-9)
Supplement: Supplementary file 1 — Accession numbers of htrA gene analyzed in this study. (DOCX 37 kb) [file 12929_2019_498_MOESM1_ESM.docx]

**Additional file 1.** Accession numbers of *htrA* gene analyzed in this study

| MH926191 | MH926192 | MH926193 | MH926194 | MH926195 |
| --- | --- | --- | --- | --- |
| MH926196 | MH926197 | MH926198 | MH926199 | MH926200 |
| MH926201 | MH926202 | MH926203 | MH926204 | MH926205 |
| MH926206 | MH926207 | MH926208 | MH926209 | MH926210 |
| MH926211 | MH926212 | MH926213 | MH926214 | MH926215 |
| MH926216 | MH926217 | MH926218 | MH926219 | MH926220 |
| MH926221 | MH926222 | MH926223 | MH926224 | MH926225 |
| MH926226 | MH926227 | MH926228 | MH926229 | MH926230 |
| MH926231 | MH926232 | MH926233 | MH926234 | MH926235 |
| MH926236 | MH926237 | MH926238 | MH926239 | MH926240 |
| MH926241 | MH926242 | MH926243 | MH926244 | MH926245 |
| MH926246 | MH926247 | MH926248 | MH926249 | MH926250 |
| MH926251 | MH926252 | MH926253 | MH926254 | MH926255 |
| MH926256 | MH926257 | MH926258 | MH926259 | MH926260 |
| MH926261 | MH926262 | MH926263 | MH926264 | MH926265 |
| MH926266 | MH926267 | MH926268 | MH926269 | MH926270 |
| MH926271 | MH926272 | MH926273 | MH926274 | MH926275 |
| MH926276 | MH926277 | MH926278 | MH926279 | MH926280 |
| MH926281 | MH926282 | MH926283 | MH926284 | MH926285 |
| MH926286 | MH926287 | MH926288 | MH926289 | MH926290 |
| MH926291 | MH926292 | MH926293 | MH926294 | MH926295 |
| MH926296 | MH926297 | MH926298 | MH926299 | MH926300 |
| MH926301 | MH926302 | MH926303 | MH926304 | MH926305 |
| MH926306 | MH926307 | MH926308 | MH926309 | MH926310 |
| MH926311 | MH926312 | MH926313 | MH926314 | MH926315 |
| MH926316 | MH926317 | MH926318 | MH926319 | MH926320 |
| MH926321 | MH926322 | MH926323 | MH926324 | MH926325 |
| MH926326 | MH926327 | MH926328 | MH926329 | MH926330 |
| MH926331 | MH926332 | MH926333 | MH926334 | MH926335 |
| MH926336 | MH926337 | MH926338 | MH926339 | MH926340 |
| MH926341 | MH926342 | MH926343 | MH926344 | MH926345 |
| MH926346 | MH926347 | MH926348 | MH926349 | MH926350 |
| MH926351 | MH926352 | MH926353 | MH926354 |  |
