# Supplementary material for: H. pylori isolates with amino acid sequence polymorphisms as presence of both HtrA-L171 & CagL-Y58/E59 increase the risk of gastric cancer
Source: J Biomed Sci. 2019 Jan 5;26:4. doi: 10.1186/s12929-019-0498-9 (PMC6321681; doi:10.1186/s12929-019-0498-9)
Supplement: Supplementary file 2 — Accession numbers of cagL gene analyzed in this study. (DOCX 28 kb) [file 12929_2019_498_MOESM2_ESM.docx]

**Additional file 2.** Accession numbers of *cagL* gene analyzed in this study

| MK014305 | MK014307 | MK014308 | MK014312 | MK014313 |
| --- | --- | --- | --- | --- |
| MK014318 | MK014323 | MK014325 | MK014333 | MK014334 |
| MK014336 | MK014338 | MK014339 | MK014340 | MK014343 |
| MK014345 | MK014346 | MK014347 | MK014349 | MK014352 |
| MK014353 | MK014355 | MK014358 | MK014359 | MK014360 |
| MK014361 | MK014364 | MK014365 | MK014368 | MK014371 |
| MK014372 | MK014374 | MK014375 | MK014376 | MK014379 |
| MK014380 | MK014382 | MK014386 | MK014387 | MK014388 |
| MK014389 | MK014390 | MK014391 | MK014394 | MK014397 |
| MK014398 | MK014400 | MK014404 | MK014409 | MK014410 |
| MK014418 | MK014419 | MK014421 | MK014422 | MK014423 |
| MK014426 | MK014428 | MK014431 | MK014432 | MK014433 |
| MK014434 | MK014435 | MK014436 | MK014437 | MK014438 |
| MK014439 | MK014440 | MK014441 | MK014442 | MK014443 |
| MK014444 | MK014445 | MK014447 | MK014451 | MK014452 |
| MK014453 | MK014454 | MK014455 | MK014456 | MK014458 |
| MK014459 | MK014460 | MK014462 | MK014463 | MK014464 |
| MK014465 | MK014466 | MK014467 | MK014468 | MK014469 |
| MK014470 | MK014471 | MK014472 | MK014474 | MK014476 |
| MK014477 | MK014478 |  |  |  |
